# Dental Anxiety among Preschool Children Attending a Tertiary Children’s Hospital: Associations with Previous Dental Experience, Parental Dental Anxiety, and Oral Health Indicators

**DOI:** 10.3290/j.ohpd.c_2842

**Published:** 2026-09-08

**Authors:** Yue Lv, Jiuying Li, Guoxia Yu

**Affiliations:** a Yue Lv Dentist, Department of Stomatology, Beijing Children’s Hospital, Capital Medical University, Beijing, China. Designed the study, collected and analyzed the data, and interpreted the findings, drafted and revised the manuscript, read and approved the final manuscript.; b Jiuying Li Dentist, Department of Stomatology, Beijing Children’s Hospital, Capital Medical University, Beijing, China. Designed the study, collected and analyzed the data, and interpreted the findings, read and approved the final manuscript.; c Guoxia Yu Chief Dentist, Department of Stomatology, Beijing Children’s Hospital, Capital Medical University, Beijing, China. Drafted and revised the manuscript, read and approved the final manuscript.

**Keywords:** child, dental anxiety, dental caries, oral hygiene, parents, preschools.

## Abstract

**Purpose:**

To estimate the prevalence of child dental anxiety (CDA) and examine associations with previous dental experience, parental dental anxiety, caries, and oral hygiene among preschool children attending a tertiary children’s hospital.

**Materials and Methods:**

This cross-sectional study included 304 children aged 3–6 years. Parents completed the Children’s Fear Survey Schedule-Dental Subscale (CFSS-DS) and Modified Dental Anxiety Scale (MDAS) and reported previous dental experience. CDA was defined as CFSS-DS ≥38. Caries and oral hygiene were assessed using the decayed, missing, and filled primary teeth (dmft) index and Simplified Oral Hygiene Index (OHI-S). Multivariable logistic regression was performed.

**Results**: CDA was identified in 148 children (48.7%); receiving an injection was the item most frequently rated as fearful. Negative previous dental experience was associated with higher odds of CDA than no prior dental visit (adjusted odds ratio [aOR] = 6.41, 95% CI: 2.86–14.34). Greater dmft was associated with higher odds (per unit; aOR = 1.27, 95% CI: 1.15–1.41), whereas older age was associated with lower odds (per year; aOR = 0.48, 95% CI: 0.35–0.67). Parental education at master’s level or above was also associated with lower odds. Adjusted linear associations of parental MDAS and OHI-S were not statistically significant.

**Conclusion:**

CDA was common in this hospital-based preschool sample. Negative previous dental experience and greater caries experience were associated with higher odds, whereas older age was associated with lower odds. Dental history and caries assessment may support individualized treatment preparation.

Child dental anxiety (CDA) is a common concern in pediatric dentistry.^5,10^ It can complicate examination and treatment when fear limits a child’s willingness to communicate or cooperate.^11^ Dental fear has also been associated with delayed attendance and poorer oral health, which may reinforce fear over time.^2^ This concern is particularly relevant in preschool children, who may have difficulty understanding unfamiliar procedures and regulating distress during dental care.^11^

Reported estimates of CDA prevalence vary widely. Differences in age, assessment instrument, cutoff value, respondent, cultural context, and study setting may contribute to this variation.^5,10^ A recent systematic review estimated that the pooled prevalence of dental fear and anxiety among young children worldwide was approximately one-third, although substantial heterogeneity was observed across studies.^18^ Preschool CDA has been studied across different populations and care settings.^17,20,23^ Differences in these populations and settings should be considered when comparing prevalence estimates and reported associations across studies.

Previous dental experience may be particularly relevant to how young children respond to subsequent dental care. Treatment history and distressing dental experiences have been associated with dental fear in children.^8,20^ Parental dental anxiety has also been examined as a family-related correlate of CDA, although findings have varied across studies.^3,19,22^ Parental education and other family characteristics may further influence children’s oral health-related behaviors and experiences.^4,22^ These factors are readily available during routine clinical assessment and may provide useful context when preparing preschool children for dental treatment.

Clinical oral health findings may provide additional information when evaluating CDA. Caries experience has been associated with dental fear and anxiety in young children,^18^ and relationships between dental status, previous treatment, and dental fear have also been reported in preschool populations.^20^ Oral hygiene may offer complementary information because it reflects aspects of daily preventive care and may coexist with broader differences in oral health needs. Caregiver knowledge and oral health practices have been linked to children’s oral health behaviors and caries experience.^16,25^ However, the associations of previous dental experience, parental dental anxiety, caries experience, and oral hygiene with CDA have been less clearly described when these factors are considered together among preschool children receiving dental care in a tertiary children’s hospital.

The present study therefore estimated the prevalence of CDA among preschool children attending a tertiary children’s hospital and examined the associations of previous dental experience, parental dental anxiety, caries experience, and oral hygiene with CDA in a multivariable analysis. By integrating family-reported information with clinical oral health measures, the study aimed to provide clinically relevant information that may inform more individualized preparation for dental care. We hypothesized that negative previous dental experience, higher parental dental anxiety, greater caries experience, and poorer oral hygiene would be associated with higher odds of CDA.

## MATERIALS AND METHODS

### Study Design and Setting

This hospital-based cross-sectional study was conducted from May 2023 to May 2024 at the dental clinic of a tertiary children’s hospital in China providing comprehensive pediatric services. The dental clinic provides routine outpatient dental examination and treatment for children.

Ethical approval was obtained from the Ethics Committee of Beijing Children’s Hospital (Approval No. 2023-E-065-Y), and the study was conducted in accordance with the Declaration of Helsinki. Written informed consent was obtained from the parents or legal guardians of all participating children.

### Eligibility Criteria

Children were eligible if they were 3 to 6 years old, attended the dental clinic during the study period, and were accompanied by a parent or legal guardian who could complete the questionnaire and provide written informed consent.

Children were excluded if they had a systemic disease or neurodevelopmental disorder that substantially interfered with completion of the clinical examination, or if they presented with an acute dental emergency that precluded questionnaire completion. Children with incomplete questionnaire or clinical examination data were also excluded.

### Participant Recruitment

Children aged 3–6 years were identified before their parents or legal guardians were approached for participation. For children with repeat attendances during the recruitment period, only the first eligible visit was retained.

A formal a-priori sample size calculation was not performed. Recruitment was conducted over the predefined study period from May 2023 to May 2024, and all eligible children who completed both the questionnaire and clinical oral examination during this period were included.

### Questionnaire Survey

Before the child underwent dental examination or treatment, the accompanying parent or legal guardian completed a paper questionnaire in the waiting area. The questionnaire collected the child’s sociodemographic information and previous dental experience and included assessments of child dental anxiety and the respondent’s own dental anxiety.

Completed questionnaires were returned to the examiner but were not reviewed before the clinical examination. The examiner was therefore unaware of the child’s Children’s Fear Survey Schedule-Dental Subscale (CFSS-DS) score, the respondent’s Modified Dental Anxiety Scale (MDAS) score, and the reported previous dental experience when recording the clinical oral health findings. Questionnaire responses and clinical examination data were entered into the study database after completion of the day’s clinical work.

### Assessment of Dental Anxiety

Child dental anxiety was assessed using the Chinese version of the Children’s Fear Survey Schedule-Dental Subscale (CFSS-DS).^12,15^ The CFSS-DS was selected because a validated Chinese version was available^12^ and its use as a parental proxy measure has been evaluated in Chinese children within the relevant age range.^15^ The accompanying parent or legal guardian completed the scale to provide a proxy assessment of the child’s dental anxiety.^15^

The CFSS-DS consists of 15 items addressing common dental situations and feared stimuli. Each item is scored from 1, indicating not afraid, to 5, indicating extremely afraid. Total scores range from 15 to 75, with higher scores indicating greater dental anxiety. A total CFSS-DS score of 38 or higher was used to define CDA.^8^

The dental anxiety of the accompanying parent or legal guardian, hereafter referred to as parental dental anxiety, was assessed using the validated Chinese version of the Modified Dental Anxiety Scale (MDAS).^24^ The MDAS was selected because its Chinese version has demonstrated good psychometric properties and provides a brief assessment of adult dental anxiety.^24^ The MDAS contains five items concerning anticipated dental visits and anxiety related to dental procedures. Each item is scored from 1 to 5, producing a total score ranging from 5 to 25. Higher scores indicate greater dental anxiety. The total MDAS score was analyzed as a continuous variable.

### Sociodemographic Characteristics and Previous Dental Experience

The questionnaire recorded the child’s age and sex. Parental education was defined as the highest educational level attained by either parent.

The accompanying parent or legal guardian provided an overall account of the child’s previous dental visits. Responses were classified as no prior dental visit, positive previous dental experience, or negative previous dental experience. Children who had never undergone a dental examination or treatment were classified as having no prior dental visit.

Previous dental experience was classified as positive when the child was described as generally calm and cooperative, without marked distress during examination or treatment. It was classified as negative when persistent crying, physical struggling, refusal to open the mouth, noticeable pain, use of physical restraint, or early termination of a visit because of severe distress was reported. When both positive and negative experiences were reported, the child was assigned to the negative previous dental experience group.

### Oral Health Assessment

All clinical oral examinations were performed by a single pediatric dentist under the operating light of a dental treatment unit. Formal intra-examiner reliability was not assessed. Oral hygiene was assessed before tooth cleaning using a modification of the Simplified Oral Hygiene Index (OHI-S) for the primary dentition.^9,14^ Six index tooth surfaces were examined: the buccal surfaces of the maxillary right and left second primary molars (teeth 55 and 65), the labial surfaces of the maxillary right and mandibular left primary central incisors (teeth 51 and 71), and the lingual surfaces of the mandibular left and right second primary molars (teeth 75 and 85). If a designated index tooth was missing or insufficiently erupted, an appropriate fully erupted substitute tooth was examined according to the established criteria for the primary-dentition modification of the OHI-S.

Debris and calculus were scored separately from 0 to 3 on each examined surface. The Simplified Debris Index (DI-S) and Simplified Calculus Index (CI-S) were calculated by dividing the respective total scores by the number of surfaces examined. The OHI-S score was calculated as the sum of the DI-S and CI-S scores and ranged from 0 to 6.

After oral hygiene assessment, the tooth surfaces were cleaned using sterile cotton rolls and air-dried with a three-way syringe to facilitate visual caries assessment. Caries status was determined primarily by visual examination using diagnostic criteria from the fifth edition of the World Health Organization Oral Health Surveys: Basic Methods.^21^ A primary tooth was recorded as decayed when a clearly cavitated carious lesion was present. Non-cavitated enamel changes were not recorded as decayed. A restored tooth without recurrent caries was recorded as filled, whereas a restored tooth with recurrent caries was recorded as decayed. A missing primary tooth was classified as missing because of caries on the basis of the child’s age, tooth position, caregiver report, or history of dental extraction. The dmft index was calculated as the sum of decayed, missing because of caries, and filled primary teeth.

### Variables and Measurements

The primary outcome was CDA, defined as a parent-reported CFSS-DS total score of 38 or higher.^8^ Children with scores below 38 were classified as not having CDA, and the outcome was analyzed as a binary variable.

For descriptive summaries of individual CFSS-DS items, responses of 3 or higher were grouped for descriptive reporting. Age, parental MDAS score, dmft index, and OHI-S score were analyzed as continuous variables. Parental education was categorized as high school or below, bachelor’s degree, or master’s degree or above. Previous dental experience was categorized as no prior dental visit, positive previous dental experience, or negative previous dental experience. Sex was treated as a categorical variable and examined in a sensitivity analysis.

For descriptive reporting, oral hygiene status was categorized according to established OHI-S criteria as good (0.0–1.2), fair (1.3–3.0), or poor (3.1–6.0). These categories were used only for descriptive analyses; the continuous OHI-S score was used in the primary regression model.

### Data Management and Statistical Analysis

Questionnaire responses and clinical examination findings were entered into an electronic spreadsheet and checked for completeness, allowable ranges, and internal consistency. Statistical analyses were performed using IBM SPSS Statistics version 26.0 (IBM; Armonk, NY, USA). Children who completed both the questionnaire and clinical oral examination were included in the analysis. No values were missing for the variables analyzed among these participants.

Categorical variables were summarized as frequencies and percentages, and continuous variables as means and standard deviations. Distributional characteristics of continuous variables were assessed using the Kolmogorov-Smirnov test together with visual inspection. Differences between children with and without CDA were examined using Pearson’s chi-squared test or Fisher’s exact test for categorical variables and the independent-samples t-test or Mann-Whitney U test for continuous variables, as appropriate. Age groups were used only for descriptive comparisons, whereas age was analyzed as a continuous variable in regression models.

Binary logistic regression was used to examine factors associated with CDA. Univariable models were first fitted to estimate unadjusted odds ratios. Variables included in the primary multivariable model were chosen on the basis of the study hypothesis and clinical relevance rather than univariable statistical significance. The primary model included age, parental education, previous dental experience, parental MDAS score, dmft index, and OHI-S score. Age, parental MDAS score, dmft index, and OHI-S score were entered as continuous variables. Parental education and previous dental experience were entered as categorical variables, with high school education or below and no prior dental visit as the respective reference categories. All variables in the primary model were entered simultaneously.

The linearity of continuous variables in the log odds was examined using Box-Tidwell transformation terms. For variables that could take a value of zero, a constant of 1 was added before logarithmic transformation. Age and dmft showed no material evidence of departure from linearity. Because parental MDAS and OHI-S showed evidence of nonlinearity, an exploratory sensitivity model containing linear and quadratic terms for both variables was fitted. Their overall contributions were assessed using likelihood-ratio tests. A separate sensitivity analysis added sex to the primary multivariable model to assess whether its inclusion materially altered the main estimates.

Univariable results were reported as odds ratios (ORs), and multivariable results as adjusted odds ratios (aORs), with 95% confidence intervals. Multicollinearity was assessed using variance inflation factors, with values below 5 considered to indicate no serious multicollinearity. Overall model fit was assessed using the Hosmer-Lemeshow goodness-of-fit test. All statistical tests were two-sided, and p < 0.05 was considered statistically significant.

## RESULTS

The datasets used and/or analyzed during the current study are available from the corresponding author on reasonable request.

### Participant Characteristics

During the study period, 385 age-eligible clinic attendances entered screening and recruitment. Of these, 25 did not meet the remaining eligibility criteria, parents or legal guardians of 18 children declined participation, and 16 children did not complete the questionnaire or clinical oral examination. In addition, 22 repeat attendances were excluded because only the first eligible visit for each child was retained. A total of 304 children completed all study assessments and were included in the final analysis. Their mean age was 4.40 ± 1.09 years. The sample included 151 boys (49.7%) and 153 girls (50.3%). By age group, 81 children (26.6%) were 3 years old, 83 (27.3%) were 4 years old, 76 (25.0%) were 5 years old, and 64 (21.1%) were 6 years old.

The highest educational level attained by either parent was a bachelor’s degree for 175 children (57.6%). A master’s degree or above was reported for 103 children (33.9%), while 26 children (8.6%) had a parental education level of high school or below. No prior dental visit was reported for 118 children (38.8%). Positive previous dental experience was reported for 91 children (29.9%), and 95 children (31.3%) were classified as having negative previous dental experience (Table 1).

**Table 1:** Characteristics of participating preschool children (N = 304)

Variable	n	%
Age group		
3 years	81	26.6
4 years	83	27.3
5 years	76	25.0
6 years	64	21.1
Sex		
Male	151	49.7
Female	153	50.3
Parental education level		
High school or below	26	8.6
Bachelor’s degree	175	57.6
Master’s degree or above	103	33.9
Previous dental experience		
No prior dental visit	118	38.8
Positive previous dental experience	91	29.9
Negative previous dental experience	95	31.3


### Oral Health Status

Most children were classified as having fair oral hygiene (OHI-S score 1.3-3.0), comprising 270 participants (88.8%). Good oral hygiene (OHI-S score 0.0-1.2) was recorded in 27 children (8.9%), whereas 7 children (2.3%) were classified as having poor oral hygiene (OHI-S score 3.1-6.0). The mean OHI-S score was 1.60 ± 0.51. The mean DI-S and CI-S scores were 1.56 ± 0.48 and 0.05 ± 0.11, respectively. The mean dmft index was 8.80 ± 4.02 (Table 2).

**Table 2:** Oral health characteristics of participating preschool children (N = 304)

Variable	Value
Oral hygiene status, n (%)	
Good (0.0–1.2)	27 (8.9)
Fair (1.3–3.0)	270 (88.8)
Poor (3.1–6.0)	7 (2.3)
OHI-S score, mean ± SD	1.60 ± 0.51
DI-S score, mean ± SD	1.56 ± 0.48
CI-S score, mean ± SD	0.05 ± 0.11
dmft index, mean ± SD	8.80 ± 4.02
OHI-S: Simplified Oral Hygiene Index; SD: standard deviation; DI-S: Simplified Debris Index; CI-S: Simplified Calculus Index; dmft: decayed, missing, and filled primary teeth.	

### Prevalence of Child Dental Anxiety and CFSS-DS Item Responses

The mean CFSS-DS score was 37.63 ± 11.59. A total of 148 children had a score of 38 or higher, corresponding to a CDA prevalence of 48.7%. The remaining 156 children (51.3%) were classified as not having CDA. The mean parental MDAS score was 7.63 ± 1.66.

In descriptive analyses, CDA prevalence differed across age groups, parental education levels, previous dental experience categories, and oral hygiene categories (all p < 0.001). No statistically significant difference was observed according to sex (p = 0.568).

CDA prevalence decreased across age groups, from 67.9% among 3-year-old children to 25.0% among 6-year-old children. Within the previous dental experience categories, the highest prevalence was observed among children with negative previous dental experience (80.0%). CDA was identified in 11.1% of children with good oral hygiene, 51.5% of those with fair oral hygiene, and 85.7% of those with poor oral hygiene. Across parental education categories, the lowest prevalence was observed among children whose parents had attained a master’s degree or above (25.2%) (Table 3).

**Table 3:** Prevalence of CDA according to demographic characteristics, previous dental experience, and oral hygiene status (N = 304)

Variable	CFSS-DS score ≥38		CFSS-DS score <38		p-value
	n	%	n	%	
Total	148	48.7	156	51.3	
Age					<0.001
3 years	55	67.9	26	32.1	
4 years	45	54.2	38	45.8	
5 years	32	42.1	44	57.9	
6 years	16	25.0	48	75.0	
Sex					0.568
Male	76	50.3	75	49.7	
Female	72	47.1	81	52.9	
Parental education level					<0.001
High school or below	19	73.1	7	26.9	
Bachelor’s degree	103	58.9	72	41.1	
Master’s degree or above	26	25.2	77	74.8	
Previous dental experience					<0.001
No prior dental visit	48	40.7	70	59.3	
Positive previous dental experience	24	26.4	67	73.6	
Negative previous dental experience	76	80.0	19	20.0	
Oral hygiene status (OHI-S)					<0.001
Good (0.0–1.2)	3	11.1	24	88.9	
Fair (1.3–3.0)	139	51.5	131	48.5	
Poor (3.1–6.0)	6	85.7	1	14.3	
CDA: child dental anxiety; CFSS-DS: Children’s Fear Survey Schedule-Dental Subscale; OHI-S: Simplified Oral Hygiene Index. p-values were obtained using Pearson’s chi-squared test, except for oral hygiene status, for which Fisher’s exact test was used.					

Among the individual CFSS-DS items, receiving an injection was most frequently assigned a score of 3 or higher, reported for 265 children (87.2%). Tooth drilling by a dentist was reported for 246 children (80.9%), followed by hearing the sound of dental drilling in 211 children (69.4%). The complete distribution of CFSS-DS item responses is presented in Table 4.

**Table 4:** Distribution of CFSS-DS item scores of 3 or higher (N = 304)

CFSS-DS item	Score ≥ 3, n (%)
Receiving an injection	265 (87.2)
Tooth drilling by a dentist	246 (80.9)
Hearing the sound of dental drilling	211 (69.4)
Choking or gagging	205 (67.4)
Watching the dentist drill	170 (55.9)
Going to a hospital	166 (54.6)
Dental instruments being placed in the mouth	128 (42.1)
Oral examination by a dentist	109 (35.9)
Opening the mouth	96 (31.6)
People wearing white uniforms	84 (27.6)
Teeth being brushed by a dentist	60 (19.7)
Physician	58 (19.1)
Dentist	42 (13.8)
Physical contact from a dentist	37 (12.2)
Being viewed by a dentist	34 (11.2)
CFSS-DS: Children’s Fear Survey Schedule-Dental Subscale. Scores of 3 to 5 were summarized as item scores of 3 or higher.	

### Univariable and Multivariable Logistic Regression Analyses

In univariable analyses, older age and parental education at master’s degree level or above were associated with lower odds of CDA. Positive previous dental experience was also associated with lower odds, whereas negative previous dental experience, higher parental MDAS, greater dmft, and higher OHI-S scores were associated with higher odds of CDA (Table 5).

**Table 5:** Univariable and multivariable logistic regression analyses of factors associated with CDA (N = 304)

Characteristic	Univariable analysis		Multivariable analysis	
	OR (95% CI)	p-value	aOR (95% CI)	p-value
Age, per 1-year increase	0.55 (0.44–0.69)	<0.001	0.48 (0.35–0.67)	<0.001
Parental education level				
High school or below	1.00 (Reference)		1.00 (Reference)	
Bachelor’s degree	0.53 (0.21–1.32)	0.171	1.18 (0.36–3.91)	0.784
Master’s degree or above	0.12 (0.05–0.33)	<0.001	0.23 (0.06–0.85)	0.027
Previous dental experience				
No prior dental visit	1.00 (Reference)		1.00 (Reference)	
Positive previous dental experience	0.52 (0.29–0.95)	0.032	0.59 (0.28–1.23)	0.162
Negative previous dental experience	5.83 (3.13–10.87)	<0.001	6.41 (2.86–14.34)	<0.001
Parental MDAS score, per 1-point increase	1.51 (1.29–1.78)	<0.001	1.28 (0.99–1.64)	0.058
dmft index, per 1-unit increase	1.32 (1.22–1.42)	<0.001	1.27 (1.15–1.41)	<0.001
OHI-S score, per 1-point increase	4.39 (2.20–8.76)	<0.001	2.43 (0.94–6.24)	0.066
CDA: child dental anxiety; OR: odds ratio; CI: confidence interval; aOR: adjusted odds ratio; MDAS: Modified Dental Anxiety Scale; dmft: decayed, missing, and filled primary teeth; OHI-S: Simplified Oral Hygiene Index; Ref: reference category. The primary multivariable model included age, parental education, previous dental experience, parental MDAS score, dmft index, and continuous OHI-S score.				

In the primary multivariable model, each additional year of age was associated with lower odds of CDA (aOR = 0.48, 95% CI: 0.35 to 0.67, p < 0.001). Parental education at master’s degree level or above was also associated with lower odds compared with high school education or below (aOR = 0.23, 95% CI: 0.06 to 0.85, p = 0.027). The bachelor’s degree category was not associated with CDA after adjustment.

Negative previous dental experience was associated with higher odds of CDA compared with no previous dental visit (aOR = 6.41, 95% CI: 2.86 to 14.34, p < 0.001). Each one-unit increase in dmft was associated with higher odds of CDA (aOR = 1.27, 95% CI: 1.15 to 1.41, p < 0.001). Positive previous dental experience was no longer statistically significant after adjustment (aOR = 0.59, 95% CI: 0.28 to 1.23, p = 0.162).

The adjusted linear associations of parental MDAS and OHI-S with CDA did not reach statistical significance in the primary model. The aOR was 1.28 per MDAS point (95% CI: 0.99 to 1.64, p = 0.058) and 2.43 per OHI-S point (95% CI: 0.94 to 6.24, p = 0.066) (Table 5).

### Model Diagnostics and Sensitivity Analyses

Variance inflation factors ranged from 1.13 to 3.90, indicating no serious multicollinearity among the variables included in the primary model. The Hosmer-Lemeshow goodness-of-fit test did not indicate evidence of lack of fit (χ^2^ = 6.62, df = 8, p = 0.578).

Adding sex to the primary multivariable model did not materially alter the principal estimates. Female sex was not associated with CDA after adjustment (aOR = 0.86, 95% CI: 0.45 to 1.62, p = 0.635).

No solid evidence of departure from linearity in the log odds was observed for age or dmft. Evidence of departure from linearity was observed for parental MDAS and OHI-S. In the exploratory sensitivity model, likelihood-ratio tests indicated statistically significant overall contributions of the combined linear and quadratic terms for parental MDAS and OHI-S (both p < 0.001). The directions of the associations for age, negative previous dental experience, and dmft remained consistent with those observed in the primary model.

## DISCUSSION

This hospital-based cross-sectional study found that nearly half of the participating preschool children met the CFSS-DS criterion for CDA. In the primary multivariable model, younger age, parent-reported negative previous dental experience, and greater caries experience were associated with higher odds of CDA, while parental education at master’s degree level or above was associated with lower odds. The adjusted linear associations of parental MDAS and OHI-S with CDA did not reach statistical significance. By considering previous dental experience, family-related factors, and clinical oral health indicators within the same analytical framework, the study provides clinically relevant information for the assessment of CDA in preschool children receiving hospital dental care.

The prevalence of CDA in the present study was higher than the pooled estimates reported in previous systematic reviews of preschool and broader pediatric populations.^10,18^ Differences in study setting may partly account for this variation. Much of the available evidence has been derived from kindergartens, schools, or community-based services, whereas children attending a tertiary hospital dental clinic may differ in their oral health needs and previous dental experiences. The mean dmft index of 8.80 in the present sample was substantially higher than that reported in population-based data from Chinese preschool children. In a national survey of 40,360 children aged 3–5 years, the mean dmft values were 2.28, 3.40, and 4.24 at ages 3, 4, and 5 years, respectively.^7^ Internationally, a recent systematic review and meta-analysis of data from 49 countries reported a pooled mean dmft of 3.68 (95% CI: 2.99–4.37) among children younger than 71 months.^13^ Although the age ranges and study populations were not identical, these comparisons indicate that caries experience was relatively high in our hospital-based sample. Because greater dmft was associated with higher odds of CDA in the multivariable analysis, the high caries burden may influence the CDA prevalence and the magnitude of the dmft-CDA association observed in this hospital-based sample. These estimates may therefore differ in community-based preschool populations with lower caries experience. Differences in age distribution, assessment instruments, cutoff values, and respondents should also be considered when comparing prevalence estimates across studies.^10,18^

The item-level findings provide further clinical context. Receiving an injection was the most frequently reported item with a CFSS-DS score of 3 or higher, followed by tooth drilling and hearing the sound of dental drilling. Drilling and injection have also been identified among the most feared dental situations in Chinese children.^8^ These findings suggest that anticipated discomfort and treatment-related sensory stimuli are particularly relevant when preparing preschool children for dental care. Recognizing these concerns before treatment may help clinicians tailor explanations and behavioral preparation to the individual child.^1,6^

Age was inversely associated with CDA. Each additional year of age was associated with lower adjusted odds of CDA, consistent with previous findings in preschool and broader pediatric populations.^8,11,23^ Developmental differences in communication, understanding of dental procedures, and emotional regulation may partly contribute to this pattern. Nevertheless, the age differences observed in this cross-sectional study should not be interpreted as evidence of within-child reductions in dental anxiety over time.

Parental education at master’s degree level or above was associated with lower odds of CDA compared with high school education or below. Higher parental education has been associated with greater oral health knowledge and more favorable child oral health behaviors.^4^ Family-related factors, including parental dental fear, have also been linked to children’s dental anxiety.^19,22^ However, the bachelor’s degree category was not associated with CDA after adjustment. The present finding therefore does not suggest a simple graded relationship between parental educational attainment and CDA and is better regarded as a secondary association that may reflect broader family circumstances.

Parent-reported negative previous dental experience showed a marked association with CDA. Children with a negative previous dental experience had substantially higher adjusted odds of CDA than those with no previous dental visit (aOR = 6.41, 95% CI: 2.86–14.34). Previous studies have similarly reported associations between distressing dental experiences, treatment history, and dental fear in children.^8,20^ Early dental encounters may shape children’s expectations of subsequent care, particularly during the preschool years. The cross-sectional design does not establish the temporal direction of this relationship, since children who are already anxious may also be more likely to experience marked distress during treatment. Nevertheless, a parent-reported history of a negative dental experience represents readily obtainable clinical information and may be particularly useful when planning communication and behavioral preparation before treatment.

Positive previous dental experience showed a different pattern. It was associated with lower odds of CDA in the univariable analysis, but the association attenuated and was no longer statistically significant after multivariable adjustment. The crude association may therefore partly reflect differences in other measured characteristics. The contrasting findings for positive and negative previous experiences also indicate that the absence of a reported negative experience should not automatically be interpreted as a protective dental history.

Caries experience remained associated with CDA after adjustment. Each one-unit increase in the dmft index was associated with 27% higher odds of CDA. This finding is consistent with previous evidence linking caries experience with dental fear and anxiety in young children.^18,20^ Greater caries experience may coexist with pain, increased treatment need, or greater exposure to restorative procedures. Dental anxiety may, in turn, complicate dental attendance and completion of treatment. Although the direction of these relationships cannot be determined from the present cross-sectional data, the persistence of the dmft association after adjustment supports consideration of caries experience alongside dental history when assessing preschool children with possible CDA.

Parental MDAS and OHI-S were both associated with CDA in the univariable analyses, but their adjusted linear associations did not reach statistical significance in the primary model. Previous studies have reported varying findings regarding the relationship between parental and child dental anxiety.^3,19,22^ The current results suggest that this relationship may be more complex than a constant increase in CDA odds with each additional MDAS point. Similarly, oral hygiene may provide useful clinical context even though the linear OHI-S association was attenuated after adjustment. In the present sample, the very low mean CI-S score indicates that the OHI-S largely reflected debris accumulation. The exploratory sensitivity analyses suggested that the relationships of parental MDAS and OHI-S with CDA may not be adequately represented by simple linear effects across their full score ranges. These findings should be regarded as exploratory and may inform further investigation in other clinical populations.

From a clinical perspective, the findings suggest that routinely available information may provide useful context when assessing and preparing preschool children with CDA for dental treatment. A parent-reported history of markedly negative dental experience and greater caries experience remained associated with higher odds of CDA after adjustment, and both can be readily identified through routine history taking and oral examination. These findings suggest that clinicians may benefit from considering previous dental experiences and caries status alongside the child’s age and dental anxiety assessment before treatment. Such information may help guide the level and content of communication and behavior guidance according to the individual child’s clinical background and previous experiences. Because these features are already available as part of routine dental assessment, their consideration does not require an additional complex screening procedure and may be readily incorporated into pretreatment planning.

This study has several strengths. It focused specifically on preschool children receiving hospital dental care, a population less well characterized than community or school-based samples. Family-reported information and previous dental experience were considered alongside clinical measures of caries and oral hygiene within the same analytical framework. All clinical oral examinations were performed using predefined criteria, and the examiner did not review questionnaire responses before completing the examination, reducing the likelihood that awareness of reported anxiety influenced the recording of oral health findings.

Several limitations should be considered. The cross-sectional design does not permit causal or temporal conclusions, and the single-center hospital-based sample may limit generalizability, particularly given the relatively high caries burden. CDA and previous dental experience were assessed through parental report and may therefore have been influenced by perception or recall. Some behaviors used to characterize a negative previous dental experience may also occur in children with pre-existing dental anxiety, which should be considered when interpreting this association. Formal intra-examiner reliability was not assessed, although all examinations were performed by the same pediatric dentist using predefined diagnostic criteria. Finally, the nonlinear findings for parental MDAS and OHI-S arose from exploratory sensitivity analyses and require confirmation in independent samples.

Taken together, the findings show that CDA is an important clinical consideration among preschool children receiving hospital dental care. Previous dental experiences and caries experience provided clinically relevant information beyond the anxiety score itself. Incorporating these readily available features into routine assessment may help clinicians recognize children who could benefit from more individualized communication and preparation before dental treatment.

## CONCLUSIONS

Child dental anxiety was common among preschool children receiving dental care at this tertiary children’s hospital. Parent-reported negative previous dental experience and greater caries experience remained associated with higher odds of CDA after adjustment, whereas older age was associated with lower odds. These findings support incorporating a brief history of previous dental experiences and routine caries assessment into pretreatment evaluation to inform more individualized communication and preparation for dental care.

## ARTIFICIAL INTELLIGENCE STATEMENT

The authors declare that no generative AI or AI-assisted technologies (such as ChatGPT, Large Language Models, or automated writing assistants) were used in the writing, editing, data analysis, or preparation of this manuscript.
